# Ammonium diamminesilver(I) bis­(5-chloro-2-hy­droxy­benzene­sulfonate) trihydrate

**DOI:** 10.1107/S160053681200339X

**Published:** 2012-01-31

**Authors:** Zhao-Peng Deng, Shan Gao, Seik Weng Ng

**Affiliations:** a Key Laboratory of Functional Inorganic Material Chemistry, Ministry of Education, Heilongjiang University, Harbin 150080, People’s Republic of China; b Department of Chemistry, University of Malaya, 50603 Kuala Lumpur, Malaysia; c Chemistry Department, Faculty of Science, King Abdulaziz University, PO Box 80203 Jeddah, Saudi Arabia

## Abstract

The reaction of silver nitrate with 5-chloro-2-hy­droxy­benzene­sulfonic acid in the presence of ammonia yielded the title salt, (NH_4_)[Ag(NH_3_)_2_](C_6_H_4_ClO_4_S)_2_·3H_2_O. The Ag^I^ ion shows linear coordination [N—Ag—N = 175.2 (1) °]. The ammonium and diamminesilver cations, the benzene­sulfonate anion and the lattice water mol­ecules inter­act through an intricate network of N—H⋯O and O—H⋯O hydrogen bonds to form a three-dimensional network.

## Related literature

For a review of metal arene­sulfonates, see: Cai (2004 ▶).
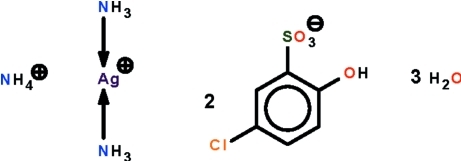


## Experimental

### 

#### Crystal data

(NH_4_)[Ag(NH_3_)_2_](C_6_H_4_ClO_4_S)_2_·3H_2_O
*M* *_r_* = 629.23
Orthorhombic, 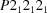

*a* = 8.8814 (8) Å
*b* = 9.8586 (10) Å
*c* = 26.434 (3) Å
*V* = 2314.5 (4) Å^3^
*Z* = 4
Mo *K*α radiation
μ = 1.34 mm^−1^
*T* = 293 K
0.19 × 0.16 × 0.13 mm

#### Data collection

Rigaku R-AXIS RAPID IP diffractometer
Absorption correction: multi-scan (*ABSCOR*; Higashi, 1995 ▶) *T*_min_ = 0.785, *T*_max_ = 0.845
22666 measured reflections
5278 independent reflections
4959 reflections with *I* > 2σ(*I*)
*R*_int_ = 0.038

#### Refinement

*R*[*F* ^2^ > 2σ(*F* ^2^)] = 0.032
*wR*(*F* ^2^) = 0.084
*S* = 1.04
5278 reflections
312 parameters
19 restraints
H atoms treated by a mixture of independent and constrained refinement
Δρ_max_ = 0.78 e Å^−3^
Δρ_min_ = −0.46 e Å^−3^
Absolute structure: Flack (1983 ▶), with 2953 Friedel pairs
Flack parameter: 0.02 (2)

### 

Data collection: *RAPID-AUTO* (Rigaku, 1998 ▶); cell refinement: *RAPID-AUTO*; data reduction: *CrystalClear* (Rigaku/MSC, 2002 ▶); program(s) used to solve structure: *SHELXS97* (Sheldrick, 2008 ▶); program(s) used to refine structure: *SHELXL97* (Sheldrick, 2008 ▶); molecular graphics: *X-SEED* (Barbour, 2001 ▶); software used to prepare material for publication: *publCIF* (Westrip, 2010 ▶).

## Supplementary Material

Crystal structure: contains datablock(s) global, I. DOI: 10.1107/S160053681200339X/bt5802sup1.cif

Structure factors: contains datablock(s) I. DOI: 10.1107/S160053681200339X/bt5802Isup2.hkl

Additional supplementary materials:  crystallographic information; 3D view; checkCIF report

## Figures and Tables

**Table 1:** Hydrogen-bond geometry (Å, °)

*D*—H⋯*A*	*D*—H	H⋯*A*	*D*⋯*A*	*D*—H⋯*A*
O4—H4⋯O3	0.84	2.38	2.970 (3)	127
O8—H8⋯O4	0.84	2.04	2.630 (2)	127
O1*w*—H11⋯O5^i^	0.84 (1)	2.00 (1)	2.838 (3)	174 (4)
O1*w*—H12⋯O3*w*	0.85 (1)	1.95 (1)	2.794 (3)	173 (4)
O2*w*—H21⋯O3	0.83 (1)	2.45 (4)	2.974 (3)	122 (4)
O2*w*—H21⋯O4	0.83 (1)	2.45 (3)	3.127 (3)	140 (5)
O2*w*—H22⋯O5^ii^	0.84 (1)	2.05 (2)	2.851 (3)	159 (5)
O3*w*—H31⋯O2^iii^	0.85 (1)	2.08 (1)	2.927 (3)	177 (5)
O3*w*—H32⋯O7^iv^	0.85 (1)	2.02 (2)	2.843 (3)	163 (5)
N1—H1*a*⋯O5	0.88	2.32	3.11 (1)	148
N1—H1*c*⋯O1^v^	0.88	2.11	2.95 (1)	158
N2—H2*a*⋯O1*w*^vi^	0.88	2.30	3.15 (1)	163
N2—H2*b*⋯O7^vii^	0.88	2.25	3.07 (1)	153
N2—H2*c*⋯O3	0.88	2.14	3.02 (1)	171
N3—H3*a*⋯O3^vi^	0.88 (1)	2.19 (1)	3.018 (3)	158 (3)
N3—H3*b*⋯O6^iv^	0.88 (1)	2.02 (1)	2.893 (3)	173 (3)
N3—H3*c*⋯O8	0.88 (1)	1.99 (1)	2.820 (3)	159 (3)
N3—H3*d*⋯O2*w*	0.88 (1)	1.94 (1)	2.826 (4)	177 (3)

Symmetry codes: (i) 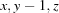; (ii) 
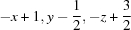
; (iii) 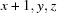; (iv) 
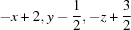
; (v) 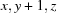; (vi) 
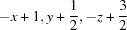
; (vii) 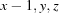.

**Fig. 1.**
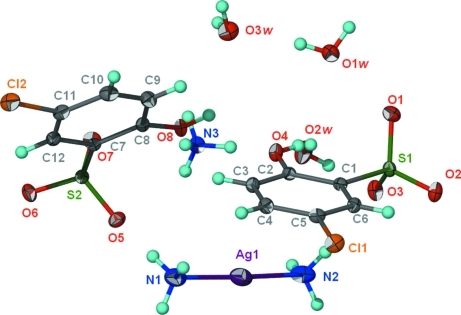
Anisotropic displacement ellipsoid plot (Barbour, 2001) of [Ag(NH3)2][NH4](C6H4ClO4S)2.3H2O at the 30% probability level; hydrogen atoms are drawn as spheres of arbitrary radius.

## supplementary crystallographic information

### Comment

Metal arenesulfonates are commonly crystalline materials; their coordination chemistry has been reviewed (Cai, 2004). In this study, the attempt to synthesize the silver(I) derivative of 5-chloro-2-hydroxybenzenesulfonic acid in the presence of ammonia gave instead the ammine-coordinated salt (Scheme I) in which the diamminesilver cation interacts indirectly with the 2-hydrox-5-chlorobenzenesulfonate anion through the coordinated ammine ligands in an outer-sphere type of coordination. In the salt, [Ag(NH_3_)_2_][NH_4_](C_6_H_4_ClO_4_S)_2_^.^3H_2_O (Fig. 1), the Ag^II^ atom shows linear coordination [N–Ag–N 175.2 (1) °]. The ammonium and diamminesilver cations, the benzenesulfonate anion and the lattice water molecules interact through N–H···O and O–H···O hydrogen bonds to form a three-dimensional network (Table 1).

### Experimental

Silver nitrate (1 mmol) and and 5-chloro-2-hydroxy-benzenesulfonic acid (1 mmol) were mixed in water (15 ml). The pH of the solution was adjusted to *ca* 6 by the addition of drops of ammonium hydroxide. The solution was filtered; colorless crystals were isolated after several days. The solution was shielded from light during the crystallization.

### Refinement

Carbon-bound H-atoms were placed in calculated positions (C–H 0.93 Å) and were included in the refinement in the riding model approximation, with *U*(H) set to 1.2*U*(C). The hydroxy H atoms were assumed to be co-planar with the aromatic ring, and these were similarly constrained (O–H 0.84 Å) and their displacement factors were set to 1.5U_eq_(O). The amino H atoms were similarly constrained (N–H 0.88 Å) and their displacement factors were set to 1.5U_eq_(N).

The water H-atoms were located in a difference Fourier map, and were refined with distance restraints O–H 0.84±0.01 Å and H···H 1.37±0.01 Å; their temperature factors were tied by a factor of 1.5 times.

The four ammonium H-atoms were located in a difference Fourier map, and were refined with distance restraints N–H 0.88±0.01 Å and H···H 1.43±0.01 Å; their temperature factors were displacement factors were set to 1.5U_eq_(O).

### Crystal data

**Table:**

(NH_4_)[Ag(NH_3_)_2_](C_6_H_4_ClO_4_S)_2_·3H_2_O	*F*(000) = 1272
*M**_r_* = 629.23	*D*_x_ = 1.806 Mg m^−^^3^
Orthorhombic, *P*2_1_2_1_2_1_	Mo *K*α radiation, λ = 0.71073 Å
Hall symbol: P 2ac 2ab	Cell parameters from 16668 reflections
*a* = 8.8814 (8) Å	θ = 3.1–27.4°
*b* = 9.8586 (10) Å	µ = 1.34 mm^−^^1^
*c* = 26.434 (3) Å	*T* = 293 K
*V* = 2314.5 (4) Å^3^	Prism, colorless
*Z* = 4	0.19 × 0.16 × 0.13 mm

### Data collection

**Table:**

Rigaku R-AXIS RAPID IP diffractometer	5278 independent reflections
Radiation source: fine-focus sealed tube	4959 reflections with *I* > 2σ(*I*)
graphite	*R*_int_ = 0.038
ω scan	θ_max_ = 27.4°, θ_min_ = 3.1°
Absorption correction: multi-scan (*ABSCOR*; Higashi, 1995)	*h* = −11→11
*T*_min_ = 0.785, *T*_max_ = 0.845	*k* = −12→12
22666 measured reflections	*l* = −32→34

### Refinement

**Table:**

Refinement on *F*^2^	Secondary atom site location: difference Fourier map
Least-squares matrix: full	Hydrogen site location: inferred from neighbouring sites
*R*[*F*^2^ > 2σ(*F*^2^)] = 0.032	H atoms treated by a mixture of independent and constrained refinement
*wR*(*F*^2^) = 0.084	*w* = 1/[σ^2^(*F*_o_^2^) + (0.0556*P*)^2^ + 0.1112*P*] where *P* = (*F*_o_^2^ + 2*F*_c_^2^)/3
*S* = 1.04	(Δ/σ)_max_ = 0.001
5278 reflections	Δρ_max_ = 0.78 e Å^−^^3^
312 parameters	Δρ_min_ = −0.46 e Å^−^^3^
19 restraints	Absolute structure: Flack (1983), with 2953 Friedel pairs
Primary atom site location: structure-invariant direct methods	Flack parameter: 0.02 (2)

### Fractional atomic coordinates and isotropic or equivalent isotropic displacement parameters (Å^2^)

**Table:**

	*x*	*y*	*z*	*U*_iso_*/*U*_eq_	
Ag1	0.37725 (3)	0.63955 (3)	0.851526 (11)	0.06155 (10)	
Cl1	0.26926 (8)	0.39184 (8)	1.06066 (2)	0.04433 (17)	
Cl2	1.22944 (8)	0.75917 (9)	0.98804 (3)	0.04893 (18)	
S1	0.30723 (6)	0.15274 (7)	0.87729 (2)	0.03079 (13)	
S2	0.91696 (6)	0.76351 (6)	0.81204 (2)	0.02806 (12)	
O1	0.4208 (2)	0.0468 (2)	0.87427 (9)	0.0498 (5)	
O2	0.1652 (2)	0.1060 (2)	0.89819 (7)	0.0425 (5)	
O3	0.2877 (2)	0.2202 (2)	0.82846 (7)	0.0422 (5)	
O4	0.5744 (2)	0.3420 (2)	0.86422 (7)	0.0358 (4)	
H4	0.5422	0.2865	0.8426	0.054*	
O5	0.75295 (19)	0.7749 (2)	0.81090 (7)	0.0402 (4)	
O6	0.9903 (2)	0.8954 (2)	0.81310 (8)	0.0414 (4)	
O7	0.9735 (2)	0.6777 (2)	0.77132 (6)	0.0392 (5)	
O8	0.8214 (2)	0.48134 (19)	0.84662 (7)	0.0335 (4)	
H8	0.7913	0.4028	0.8537	0.050*	
O1w	0.6740 (2)	0.0537 (2)	0.81430 (10)	0.0506 (5)	
H11	0.692 (4)	−0.0301 (11)	0.8143 (19)	0.076*	
H12	0.756 (3)	0.095 (3)	0.8202 (18)	0.076*	
O2w	0.5127 (3)	0.2905 (3)	0.74936 (9)	0.0592 (6)	
H21	0.516 (5)	0.262 (6)	0.7788 (8)	0.089*	
H22	0.433 (3)	0.266 (6)	0.7351 (14)	0.089*	
O3w	0.9314 (3)	0.2107 (3)	0.83062 (9)	0.0526 (6)	
H31	0.998 (3)	0.177 (4)	0.8501 (11)	0.079*	
H32	0.970 (4)	0.216 (5)	0.8012 (7)	0.079*	
N1	0.5129 (3)	0.7659 (3)	0.89623 (12)	0.0617 (8)	
H1A	0.6036	0.7708	0.8829	0.093*	
H1B	0.5191	0.7323	0.9270	0.093*	
H1C	0.4730	0.8475	0.8975	0.093*	
N2	0.2539 (4)	0.5159 (4)	0.80167 (12)	0.0650 (8)	
H2A	0.2936	0.5207	0.7712	0.098*	
H2B	0.1599	0.5438	0.8006	0.098*	
H2C	0.2567	0.4314	0.8123	0.098*	
N3	0.7473 (3)	0.4833 (3)	0.74290 (9)	0.0386 (5)	
H3A	0.714 (3)	0.5551 (19)	0.7267 (10)	0.058*	
H3B	0.825 (2)	0.449 (3)	0.7270 (10)	0.058*	
H3C	0.773 (3)	0.505 (3)	0.7738 (5)	0.058*	
H3D	0.674 (2)	0.423 (2)	0.7437 (11)	0.058*	
C1	0.3775 (2)	0.2748 (2)	0.92047 (8)	0.0273 (4)	
C2	0.5039 (2)	0.3542 (3)	0.90939 (8)	0.0276 (4)	
C3	0.5558 (3)	0.4449 (3)	0.94605 (10)	0.0328 (5)	
H3	0.6390	0.4990	0.9390	0.039*	
C4	0.4859 (3)	0.4562 (3)	0.99284 (10)	0.0342 (5)	
H4A	0.5220	0.5163	1.0171	0.041*	
C5	0.3610 (3)	0.3760 (3)	1.00269 (8)	0.0316 (5)	
C6	0.3062 (3)	0.2859 (3)	0.96722 (9)	0.0310 (5)	
H6	0.2223	0.2330	0.9745	0.037*	
C7	0.9634 (2)	0.6794 (3)	0.86901 (8)	0.0261 (5)	
C8	0.9076 (3)	0.5478 (3)	0.87867 (9)	0.0289 (5)	
C9	0.9509 (3)	0.4891 (3)	0.92511 (10)	0.0381 (6)	
H9	0.9140	0.4037	0.9335	0.046*	
C10	1.0459 (3)	0.5539 (3)	0.95862 (10)	0.0393 (6)	
H10	1.0720	0.5125	0.9890	0.047*	
C11	1.1019 (3)	0.6808 (3)	0.94671 (9)	0.0347 (5)	
C12	1.0603 (3)	0.7457 (3)	0.90287 (9)	0.0318 (5)	
H12A	1.0960	0.8323	0.8957	0.038*	

### Atomic displacement parameters (Å^2^)

**Table:**

	*U*^11^	*U*^22^	*U*^33^	*U*^12^	*U*^13^	*U*^23^
Ag1	0.06878 (17)	0.05382 (16)	0.06205 (16)	0.01435 (13)	0.00984 (12)	0.00913 (13)
Cl1	0.0497 (3)	0.0586 (5)	0.0247 (2)	0.0016 (3)	0.0049 (3)	−0.0006 (3)
Cl2	0.0467 (3)	0.0593 (5)	0.0407 (3)	0.0043 (3)	−0.0153 (3)	−0.0148 (3)
S1	0.0335 (3)	0.0301 (3)	0.0288 (3)	−0.0049 (2)	0.0004 (2)	−0.0032 (2)
S2	0.0295 (3)	0.0293 (3)	0.0253 (2)	−0.0007 (2)	0.00047 (19)	0.0021 (2)
O1	0.0501 (11)	0.0354 (11)	0.0638 (13)	0.0047 (9)	−0.0003 (10)	−0.0095 (10)
O2	0.0385 (9)	0.0505 (13)	0.0386 (9)	−0.0150 (9)	0.0000 (8)	−0.0016 (9)
O3	0.0535 (10)	0.0453 (12)	0.0278 (8)	−0.0144 (9)	−0.0047 (8)	−0.0002 (8)
O4	0.0370 (8)	0.0407 (11)	0.0297 (8)	−0.0101 (8)	0.0057 (7)	−0.0058 (7)
O5	0.0310 (8)	0.0476 (12)	0.0420 (9)	0.0022 (8)	−0.0026 (7)	0.0069 (9)
O6	0.0496 (10)	0.0301 (10)	0.0446 (10)	−0.0075 (8)	−0.0031 (9)	0.0086 (8)
O7	0.0467 (10)	0.0459 (12)	0.0249 (8)	0.0035 (9)	0.0055 (7)	0.0006 (8)
O8	0.0386 (8)	0.0318 (9)	0.0303 (8)	−0.0108 (7)	0.0002 (7)	−0.0008 (7)
O1w	0.0506 (11)	0.0410 (12)	0.0604 (13)	0.0008 (10)	0.0040 (10)	−0.0035 (11)
O2w	0.0520 (12)	0.0760 (18)	0.0494 (11)	−0.0175 (13)	−0.0092 (10)	0.0148 (13)
O3w	0.0536 (12)	0.0635 (16)	0.0408 (10)	0.0040 (11)	0.0052 (9)	−0.0005 (11)
N1	0.0641 (16)	0.0554 (18)	0.0655 (17)	0.0117 (15)	0.0119 (14)	0.0201 (15)
N2	0.0724 (19)	0.065 (2)	0.0576 (17)	0.0251 (17)	0.0065 (15)	0.0041 (15)
N3	0.0414 (11)	0.0403 (13)	0.0340 (11)	−0.0020 (10)	−0.0015 (9)	0.0003 (9)
C1	0.0284 (9)	0.0276 (11)	0.0259 (10)	0.0015 (9)	−0.0031 (8)	−0.0021 (9)
C2	0.0291 (10)	0.0270 (11)	0.0267 (9)	0.0011 (9)	−0.0009 (8)	0.0007 (9)
C3	0.0321 (11)	0.0322 (13)	0.0341 (12)	−0.0035 (10)	−0.0004 (9)	−0.0022 (10)
C4	0.0390 (12)	0.0341 (14)	0.0294 (11)	−0.0021 (11)	−0.0030 (10)	−0.0033 (10)
C5	0.0348 (11)	0.0371 (14)	0.0227 (10)	0.0044 (11)	0.0015 (9)	0.0014 (9)
C6	0.0309 (10)	0.0345 (14)	0.0277 (11)	−0.0028 (10)	0.0001 (9)	0.0044 (10)
C7	0.0246 (9)	0.0320 (13)	0.0217 (9)	0.0024 (9)	0.0015 (8)	−0.0002 (8)
C8	0.0293 (10)	0.0313 (12)	0.0261 (10)	−0.0023 (9)	0.0042 (9)	−0.0004 (10)
C9	0.0445 (14)	0.0362 (14)	0.0336 (12)	−0.0007 (11)	−0.0002 (11)	0.0056 (11)
C10	0.0434 (13)	0.0457 (16)	0.0289 (11)	0.0098 (12)	−0.0050 (10)	0.0036 (11)
C11	0.0321 (11)	0.0445 (15)	0.0276 (11)	0.0024 (10)	−0.0047 (9)	−0.0094 (10)
C12	0.0297 (10)	0.0338 (13)	0.0318 (11)	0.0006 (10)	0.0003 (8)	−0.0046 (10)

### Geometric parameters (Å, °)

**Table:**

Ag1—N1	2.097 (4)	N2—H2A	0.8800
Ag1—N2	2.103 (4)	N2—H2B	0.8800
Cl1—C5	1.743 (2)	N2—H2C	0.8800
Cl2—C11	1.753 (2)	N3—H3A	0.878 (9)
S1—O1	1.454 (2)	N3—H3B	0.877 (9)
S1—O2	1.4522 (19)	N3—H3C	0.875 (9)
S1—O3	1.462 (2)	N3—H3D	0.883 (9)
S1—C1	1.772 (2)	C1—C2	1.400 (3)
S2—O6	1.454 (2)	C1—C6	1.393 (3)
S2—O7	1.4580 (19)	C2—C3	1.397 (4)
S2—O5	1.4613 (18)	C3—C4	1.388 (4)
S2—C7	1.768 (2)	C3—H3	0.9300
O4—C2	1.353 (3)	C4—C5	1.387 (4)
O4—H4	0.8400	C4—H4A	0.9300
O8—C8	1.316 (3)	C5—C6	1.380 (4)
O8—H8	0.8400	C6—H6	0.9300
O1w—H11	0.841 (10)	C7—C12	1.403 (3)
O1w—H12	0.849 (10)	C7—C8	1.413 (4)
O2w—H21	0.828 (10)	C8—C9	1.410 (4)
O2w—H22	0.839 (10)	C9—C10	1.380 (4)
O3w—H31	0.849 (10)	C9—H9	0.9300
O3w—H32	0.852 (10)	C10—C11	1.382 (4)
N1—H1A	0.8800	C10—H10	0.9300
N1—H1B	0.8800	C11—C12	1.374 (4)
N1—H1C	0.8800	C12—H12A	0.9300
			
N1—Ag1—N2	175.18 (13)	C2—C1—C6	120.4 (2)
O1—S1—O2	113.33 (13)	C2—C1—S1	121.83 (17)
O1—S1—O3	111.12 (14)	C6—C1—S1	117.72 (18)
O2—S1—O3	112.13 (12)	O4—C2—C1	120.4 (2)
O1—S1—C1	106.19 (12)	O4—C2—C3	121.1 (2)
O2—S1—C1	106.03 (11)	C1—C2—C3	118.5 (2)
O3—S1—C1	107.56 (12)	C4—C3—C2	121.4 (2)
O6—S2—O7	112.24 (12)	C4—C3—H3	119.3
O6—S2—O5	112.22 (13)	C2—C3—H3	119.3
O7—S2—O5	111.88 (12)	C3—C4—C5	118.7 (2)
O6—S2—C7	107.34 (12)	C3—C4—H4A	120.7
O7—S2—C7	106.06 (11)	C5—C4—H4A	120.7
O5—S2—C7	106.62 (11)	C4—C5—C6	121.4 (2)
C2—O4—H4	120.0	C4—C5—Cl1	119.20 (19)
C8—O8—H8	120.0	C6—C5—Cl1	119.37 (19)
H11—O1w—H12	108 (2)	C5—C6—C1	119.6 (2)
H21—O2w—H22	111 (2)	C5—C6—H6	120.2
H31—O3w—H32	107 (2)	C1—C6—H6	120.2
Ag1—N1—H1A	109.5	C12—C7—C8	121.8 (2)
Ag1—N1—H1B	109.5	C12—C7—S2	117.93 (19)
H1A—N1—H1B	109.5	C8—C7—S2	120.21 (17)
Ag1—N1—H1C	109.5	O8—C8—C9	121.0 (2)
H1A—N1—H1C	109.5	O8—C8—C7	123.0 (2)
H1B—N1—H1C	109.5	C9—C8—C7	116.0 (2)
Ag1—N2—H2A	109.5	C10—C9—C8	122.4 (3)
Ag1—N2—H2B	109.5	C10—C9—H9	118.8
H2A—N2—H2B	109.5	C8—C9—H9	118.8
Ag1—N2—H2C	109.5	C9—C10—C11	119.5 (2)
H2A—N2—H2C	109.5	C9—C10—H10	120.2
H2B—N2—H2C	109.5	C11—C10—H10	120.2
H3A—N3—H3B	110 (1)	C12—C11—C10	121.1 (2)
H3A—N3—H3C	110 (1)	C12—C11—Cl2	119.6 (2)
H3B—N3—H3C	110 (1)	C10—C11—Cl2	119.3 (2)
H3A—N3—H3D	108 (1)	C11—C12—C7	119.1 (2)
H3B—N3—H3D	110 (1)	C11—C12—H12A	120.5
H3C—N3—H3D	110 (1)	C7—C12—H12A	120.5
			
O1—S1—C1—C2	67.4 (2)	O6—S2—C7—C12	−2.1 (2)
O2—S1—C1—C2	−171.8 (2)	O7—S2—C7—C12	118.10 (19)
O3—S1—C1—C2	−51.6 (2)	O5—S2—C7—C12	−122.51 (19)
O1—S1—C1—C6	−110.0 (2)	O6—S2—C7—C8	−179.93 (18)
O2—S1—C1—C6	10.8 (2)	O7—S2—C7—C8	−59.8 (2)
O3—S1—C1—C6	130.9 (2)	O5—S2—C7—C8	59.6 (2)
C6—C1—C2—O4	179.1 (2)	C12—C7—C8—O8	−177.2 (2)
S1—C1—C2—O4	1.7 (3)	S2—C7—C8—O8	0.6 (3)
C6—C1—C2—C3	−0.4 (4)	C12—C7—C8—C9	2.2 (3)
S1—C1—C2—C3	−177.78 (19)	S2—C7—C8—C9	179.98 (18)
O4—C2—C3—C4	−178.8 (2)	O8—C8—C9—C10	177.4 (2)
C1—C2—C3—C4	0.8 (4)	C7—C8—C9—C10	−2.0 (4)
C2—C3—C4—C5	−0.7 (4)	C8—C9—C10—C11	−0.2 (4)
C3—C4—C5—C6	0.2 (4)	C9—C10—C11—C12	2.3 (4)
C3—C4—C5—Cl1	−178.5 (2)	C9—C10—C11—Cl2	−177.0 (2)
C4—C5—C6—C1	0.1 (4)	C10—C11—C12—C7	−2.2 (4)
Cl1—C5—C6—C1	178.78 (19)	Cl2—C11—C12—C7	177.22 (18)
C2—C1—C6—C5	0.0 (4)	C8—C7—C12—C11	−0.2 (3)
S1—C1—C6—C5	177.48 (19)	S2—C7—C12—C11	−178.01 (18)

### Hydrogen-bond geometry (Å, °)

**Table:**

*D*—H···*A*	*D*—H	H···*A*	*D*···*A*	*D*—H···*A*
O4—H4···O3	0.84	2.38	2.970 (3)	127
O8—H8···O4	0.84	2.04	2.630 (2)	127
O1w—H11···O5^i^	0.84 (1)	2.00 (1)	2.838 (3)	174 (4)
O1w—H12···O3w	0.85 (1)	1.95 (1)	2.794 (3)	173 (4)
O2w—H21···O3	0.83 (1)	2.45 (4)	2.974 (3)	122 (4)
O2w—H21···O4	0.83 (1)	2.45 (3)	3.127 (3)	140 (5)
O2w—H22···O5^ii^	0.84 (1)	2.05 (2)	2.851 (3)	159 (5)
O3w—H31···O2^iii^	0.85 (1)	2.08 (1)	2.927 (3)	177 (5)
O3w—H32···O7^iv^	0.85 (1)	2.02 (2)	2.843 (3)	163 (5)
N1—H1a···O5	0.88	2.32	3.11 (1)	148
N1—H1c···O1^v^	0.88	2.11	2.95 (1)	158
N2—H2a···O1w^vi^	0.88	2.30	3.15 (1)	163
N2—H2b···O7^vii^	0.88	2.25	3.07 (1)	153
N2—H2c···O3	0.88	2.14	3.02 (1)	171
N3—H3a···O3^vi^	0.88 (1)	2.19 (1)	3.018 (3)	158 (3)
N3—H3b···O6^iv^	0.88 (1)	2.02 (1)	2.893 (3)	173 (3)
N3—H3c···O8	0.88 (1)	1.99 (1)	2.820 (3)	159 (3)
N3—H3d···O2w	0.88 (1)	1.94 (1)	2.826 (4)	177 (3)

Symmetry codes: (i) *x*, *y*−1, *z*; (ii) −*x*+1, *y*−1/2, −*z*+3/2; (iii) *x*+1, *y*, *z*; (iv) −*x*+2, *y*−1/2, −*z*+3/2; (v) *x*, *y*+1, *z*; (vi) −*x*+1, *y*+1/2, −*z*+3/2; (vii) *x*−1, *y*, *z*.
